# *Sarcocystis* species in bovine carcasses from a Belgian abattoir: a cross-sectional study

**DOI:** 10.1186/s13071-021-04788-1

**Published:** 2021-05-21

**Authors:** Hang Zeng, Inge Van Damme, Teresia Wanjiru Kabi, Barbara Šoba, Sarah Gabriël

**Affiliations:** 1 Department of Veterinary Public Health and Food Safety, Faculty of Veterinary Medicine, Ghent University, Salisburylaan 133, 9820 Merelbeke, Belgium; 2 Institute of Microbiology and Immunology, Faculty of Medicine, University of Ljubljana, Zaloška 4, 1000 Ljubljana, Slovenia

**Keywords:** *Sarcocystis* spp., Cattle, Carcass, Belgium, *Sarcocystis hominis*, *Cox*1, Observational study

## Abstract

**Background:**

*Sarcocystis* species are obligatorily heteroxenous parasites, of which some are zoonotic, representing a public health and economic impact. This study investigated the occurrence of *Sarcocystis* spp. in cattle sampled from a Belgian slaughterhouse.

**Methods:**

A total of 200 carcasses were included in the study, sampled during 10 sampling days. The sedimentation method was applied to isolate the sarcocysts from both heart and diaphragm muscles collected from each carcass. Multiplex PCR, PCR–RFLP as well as *cox*1 gene sequencing techniques were applied serially on collected sarcocysts for species identification.

**Results:**

*Sarcocystis* spp. were detected in 64% (128/200; 95% CI 57–71%) of the sampled carcasses. Female dairy cattle presented the highest *Sarcocystis* occurrence rate (91%) as well as the highest *Sarcocystis* species diversity compared to female beef and male beef. *Sarcocystis* spp. were detected more often in the heart muscles than in the diaphragm among female beef (*p* < 0.001) and dairy carcasses (*p* = 0.001), while in male carcasses no significant difference was observed (*p* = 0.763). The effect of age was not significant in male carcasses (*p* = 0.872), while the odds of finding sarcocysts significantly increased with age (*p* = 0.003) within both types of female carcasses. *S. cruzi* was the most prevalent species and was found in 56.5% (113/200) of the carcasses, followed by *S. hominis* (21.0%, 42/200), *S. bovifelis* (12.5%, 25/200), *S. bovini* (2.0%, 4/200), *S. hirsuta* (1.5%, 3/200) and *S. heydorni* (0.5%, 1/200). Six different species were detected in the diaphragm, while only two species were recovered from the heart. *S. cruzi* was the most prevalent species in heart, while in the diaphragm, this was *S. hominis*.

**Conclusions:**

The detection of *S. hominis* in 21% of the sampled carcasses presents a potential food safety issue, and further research is warranted into controlling this infection.

**Graphic Abstract:**

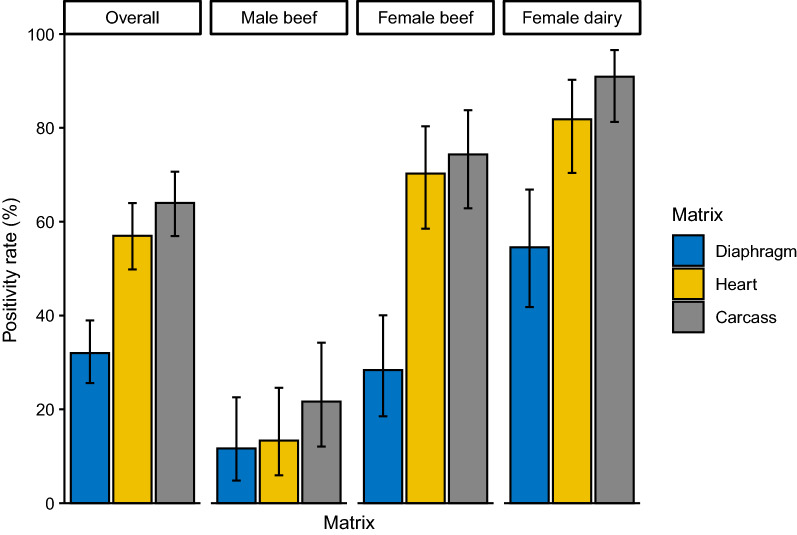

**Supplementary Information:**

The online version contains supplementary material available at 10.1186/s13071-021-04788-1.

## Background

*Sarcocystis* species (Apicomplexa: Sarcocystidae) are intracellular protozoan parasites, of which some species are zoonotic. Since their first discovery in striated muscles of a domestic mouse in 1843, over 200 *Sarcocystis* species have been identified. The complete life cycles are known for only 26 species [1]. The complete life cycle of *Sarcocystis* requires two hosts, generally a herbivore or carnivore intermediate host and a carnivore or omnivore definitive host. Cattle are the intermediate host of several *Sarcocystis* species (of which some species names are still debated), including *S. cruzi* with canids as definitive host, *S. bovifelis*, *S. bovini* and *S. hirsuta* with felids as definitive host, and *S. hominis* and *S. heydorni* with humans as definitive host [2–4]. *Sarcocystis rommeli*, which in fact is considered to be *S. bovifelis* by Gjerde [4], has felids as definitive host [5]. In the case of *S. hominis* and *S. heydorni*, intestinal sarcocystiosis may develop after consuming raw or undercooked beef containing mature sarcocysts, with symptoms such as abdominal pain, distension, watery diarrhea and eosinophilia [6]. Oocysts (mature oocyst containing two sporocysts) are formed in the small intestine and are excreted with the stool into the environment either as oocysts or when the oocyst rupture, as sporocysts [7, 8].

Bovine eosinophilic myositis (BEM) is a specific inflammatory myopathy that might be associated with *S. cruzi* and *S. hominis* infections [9–12]. Although usually no clinical symptoms are observed in animals, the gross lesions of BEM (green, focal stripes or patches) are detected in the cattle carcass after slaughter and cause carcass condemnation and economic losses [9, 13, 14]. In the study of Vangeel et al. [13], *S. hominis*, *S. cruzi* and *S. hirsuta* were detected in lesions of BEM, indicating that BEM was not only associated with one specific *Sarcocystis* species. On the contrary, not all the *Sarcocystis* infections present BEM.

*Sarcocystis* infection in cattle is reported worldwide, with an occurrence rate between 36.2% and 100% [15–20]. *Sarcocystis cruzi* and *S. hominis* are the most commonly reported species and are found in different muscles in cattle, e.g. heart, diaphragm, tongue and esophagus [21–24]. However, the results from those studies are difficult to compare as different sample types from different locations were collected, and different techniques were used to define infection status and species identification. Specifically, for Belgium, Vangeel et al. [23] found complete sarcocysts or fragments of sarcocysts in 94% of raw minced beef samples from retail stores. Molecular results revealed that 97.4% of the collected thick-walled sarcocysts were *S. hominis*. Based on the results, it was assumed that most carcasses are infected with this zoonotic species in Belgium.

Although high occurrences of *Sarcocystis* spp. in different cattle muscles, minced meat or beef burger samples have been reported, the species identification methods were not consistent and not adapted to the newly defined (though debated) *S. bovifelis*, *S. bovini* and *S. heydorni* species. In Belgium, there are no recent studies determining the occurrence of *Sarcocystis* species in slaughtered cattle. Therefore, heart and diaphragm samples were collected from carcasses in a Belgian slaughterhouse, followed by sarcocyst isolation and identification based on sedimentation and updated molecular methods. Besides, the associations between the presence, intensity and diversity of *Sarcocystis* spp. and different animal characteristics (age, type and sex) were explored.

## Methods

### Sample collection

A Belgian cattle slaughterhouse, located in Flanders—the northern part of Belgium—was visited 10 times from July to October 2019. During each visit, around 20 consecutive carcasses were sampled. Heart and diaphragm muscles (100–150 g of each) were collected from each of the 201 selected carcasses and stored in separate sealed bags. All samples were transported to the laboratory and stored at 4 °C until analysis. For all sampled carcasses, the age, category (A–E, according to Council Regulation (EC) No. 1234/2007 [25]), classification [conformation (S, E, U, R, O, P) and fat cover (1–5) class] were obtained from the slaughterhouse.

### Microscopic examination following sedimentation

A modified sedimentation method according to Vangeel et al. [23] was applied in this study. For each sample (heart and diaphragm), fat tissue and fasciae were removed, after which 100 g was minced with 30 ml of saline solution for approximately 5 s at high speed in a blender. A glass funnel ending with a rubber tube on a tripod was closed with a clip near the end of the rubber tube, with a sieve in combination with a single layer of gauze on top of the funnel. The minced meat was put on the gauze, and preheated (37 °C) saline solution was added until the minced meat was fully covered. The whole apparatus was incubated at 37 °C for 4 h. Every hour, the minced meat in the sieve was gently stirred with a spoon.

At the end of the incubation, 10 ml of the solution from the bottom of the apparatus was collected into a glass flask by opening the spring clip on the rubber tube. This sediment was examined for the presence of sarcocysts in a graded petri dish with a stereo microscope (×40). During microscopic examination, the morphology of the sarcocysts was recorded, e.g. thin-walled or thick-walled, with pointed or rounded edge [23, 26]. The microscopic examination was stopped when 15 sarcocysts were identified. Identified sarcocysts were stored separately in 1.5-ml tubes with 20 µl of saline solution. In addition, the number of identified sarcocysts was recorded. When 15 or more sarcocysts were identified, the number of sarcocysts was recorded as 15. All the sarcocysts were stored at −20 °C. From each sample, at least one sarcocyst per morphologic group (see above, e.g. thick-walled and rounded edge) was selected for molecular identification to species level.

### Molecular identification of *Sarcocystis* species

The DNA of a single sarcocyst was extracted using the Tissue and Hair Extraction Kit (Promega, USA) according to the manufacturer’s recommendations. Three techniques were applied to identify the *Sarcocystis* species. All selected sarcocysts were subjected to multiplex polymerase chain reaction (mPCR) [27], after which the unidentifiable sarcocysts were subjected to PCR-restriction fragment length polymorphism (PCR–RFLP); primers 18S9L: 5’-GGATAAACCGTGGTAATTCTATG-3’ and 18S1H: 5’-GGCAAATGCTTTCGCAGTAG-3’ were from Hamidinejat et al. [28]. Sarcocysts that could also not be identified with the latter technique were sent for sequencing. All the primers in this study were from Sigma-Aldrich (USA).

#### Multiplex PCR

The PCR was conducted according to Rubiola et al. [27], except that 1 mM instead of 0.5 mM of Sarco Rev primer was used in the PCR mixture. The PCR results were separated in 2% agarose (0.5× TAE buffer, 70 min at 120 V) and visualized using UV light. Sarcocysts with only a band at 200–250 base pairs (bp), the size of the *Sarcocystis* spp. fragment, were considered unidentified and were tested using PCR–RFLP. For sarcocysts without the 200–250-bp band nor any of the species-specific bands, the sarcocyst was recorded as *Sarcocystis*-negative.

#### PCR–RFLP

After amplification of the 18S rDNA (using primers 18S9L and 18S1H according to Hamidinejat et al. [28]), the fragments (approx. 900 bp) were digested with the *Fok*I [29] and *Bfa*I [30] enzymes. The PCR was modified from Jehle et al. [29]. The 50-µL PCR reaction mix contained 25 µL of GoTaq G2 Hot Start Master Mix (Promega), 0.5 µL of each primer (working solution: 50 pmol/µl), 19 µL of nuclease-free water and 5 µL of sample DNA. The PCR was performed with the following protocol: pre-denaturation at 94 °C for 2 min, 35 cycles of denaturation at 94 °C for 40 s, annealing at 61 °C for 60 s and elongation at 72 °C for 80 s, and a final elongation at 72 °C for 5 min. The PCR products were separated in 2% agarose (0.5× TAE -buffer, 60 min at 110 V) and visualized using UV light. Ten microliters of obtained PCR product was digested using 30 µL digestion mix containing 5U of the enzymes (*Fok*I, *Bfa*I (FastDigest, Thermo Scientific)) at 37 °C for 2 h, respectively. The results were visualized in 2.5% agarose (0.5× TAE buffer, 90 min at 120 V). The size (in bp) of the expected 18S rDNA digestion fragments for each species distinguished by agarose gel electrophoresis PCR–RFLP is shown in Additional file 1.

#### Sequencing

If the species was not identified by either the mPCR or the PCR–RFLP, then the sarcocyst was sent to Eurofins Genomics (Luxembourg) for sequencing based on the mitochondrial cytochrome c oxidase subunit I gene (*cox*1). Sarc cox1 SF and Sarc cox1 SR 9 primers were used according to Gjerde [4], and the PCR protocol was applied according to Hoeve-Bakker et al. [24]. The sequences obtained were compared to available sequences from the GenBank database using BLAST. The nucleotide sequences of these unidentified *Sarcocystis* spp. were deposited in the GenBank database under the following accession numbers: UNS1: MW756133, UNS2: MW756134, and UNS3: MW756135.

### Statistical analysis

A prevalence of 90% was assumed to calculate the sample size, which was based on the study of Vangeel et al. [23]. At least 138 samples would be needed to ensure precision of 5% of the 95% confidence interval (CI) around the prevalence estimate. For a prevalence of 85%, 196 samples would be required to obtain the same precision. To account for a lower prevalence than initially anticipated and potential losses, a total of 201 carcasses were sampled. One carcass was classified as category Z (i.e. from an animal above 8 but below 12 months old) and was excluded from the analysis set, resulting in a final sample of 200 carcasses. All analyses were performed using R version 4.0.2 [31]. The final dataset that was used for the analyses is deposited open access in Zenodo [32].

Based on the conformation, carcasses were categorized into two types: the S/E/U conformation categories were assumed to be beef cattle, and R/O/P were categorized as dairy cattle. When including the sex of the animal, three distinct groups were created: male beef, female beef and female dairy cattle. Binomial and categorical variables were summarized using tables, continuous variables (age) were inspected visually using histograms, and count data (intensity and number of different species) using bar plots. An organ was considered positive when at least one sarcocyst was molecularly confirmed as *Sarcocystis* spp. If sarcocysts were recovered by microscopy, but results were not confirmed by molecular identification, then the organ was considered negative. A carcass was considered positive if the diaphragm and/or heart was positive. The *Sarcocystis* infection results were summarized for the different groups of cattle (male beef, female beef and female dairy cattle). The 95% Clopper–Pearson confidence intervals around binomial proportions (number of positives/total number tested) were calculated using the DescTools package [33]. The proportion of *Sarcocystis*-positive organs (heart versus diaphragm) were compared using the McNemar chi-square test for paired proportions. Differences in *Sarcocystis* positivity between the three groups were tested using logistic regressions, with the *Sarcocystis* result (positive/negative) as outcome and the group as exploratory variable. The relation between *Sarcocystis* positivity and age was assessed within males and females separately, because almost all males were younger than females. Age (in years) was included as a continuous predictor variable. For females, the type (beef/dairy) was included as main effect, and the interaction between type and age was also evaluated. The different models were compared using the Akaike information criterion (AIC), and the model with the lowest AIC (which was the model without type–age interaction) was used. To compare species diversity (expressed as the total number of unique *Sarcocystis* species within a carcass) between the different groups, nonparametric pairwise Wilcoxon Rank Sum tests were performed, adjusting for multiple testing using the Holm correction.

A phylogenetic tree was constructed using MEGA 7.0 [34] via the neighbor-joining method.

## Results

**Fig. 1:**
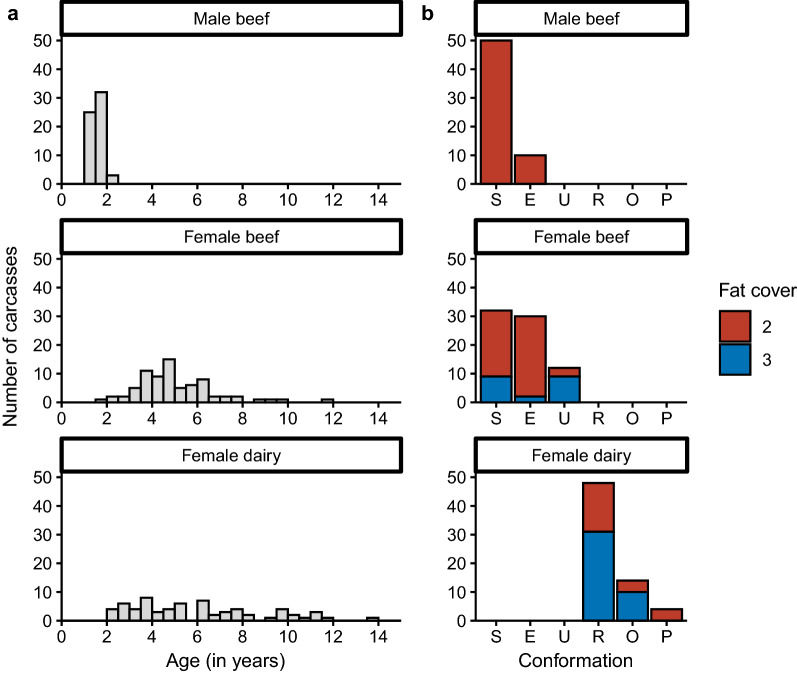
**a** Age distribution and **b** classification (conformation and fat cover) of bovine carcasses. Data are presented for each of the three different groups of carcasses: male beef (*n* = 60), female beef (*n* = 74) and female dairy (*n* = 66)

In total, the results of 200 carcasses were analyzed. Based on sex and type, carcasses originated from three distinct groups of animals: 60 male beef, 74 female beef and 66 female dairy cattle. The distribution of age, conformation and fat content within each of these groups is visualized in Fig. 1. The mean age of male beef, female beef and female dairy cattle was 1.6 years (range from 1.1 to 2.2 years), 5.1 years (1.7–11.8 years) and 6.1 years (2.1–13.6 years), respectively.

Overall, *Sarcocystis* spp. were detected in 64% (128/200; 95% CI 57–71%) of all the sampled carcasses. *Sarcocystis* spp. were found in the heart of 114 carcasses (57%, 95% CI 50–64%) and the diaphragm of 64 carcasses (32%, 95% CI 26–39%). In 50 carcasses (25%), both the heart and diaphragm were *Sarcocystis*-positive, while only the heart and diaphragm were positive in 64 and 14 carcasses, respectively. The proportion of positive heart samples was significantly higher than the diaphragm samples (McNemar’s statistic = 32, df = 1, *p* < 0.001).

### *Sarcocystis* spp. occurrence in female beef, female dairy and male beef carcasses

**Fig. 2:**
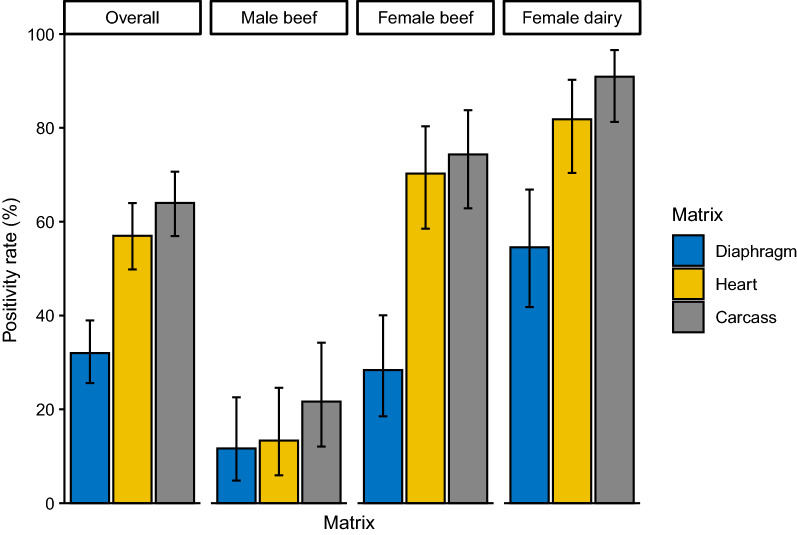
The occurrence of *Sarcocystis* spp. in cattle carcasses during slaughter. Data are presented overall (*n* = 200) and per group: male beef (*n* = 60), female beef (*n* = 74) and female dairy carcasses (*n* = 66). The error bars indicate the 95% confidence interval

Carcasses from female dairy carcasses had the highest *Sarcocystis* occurrence rate (91%; 95% CI 81–97%) among the three different groups (Fig. 2). In the beef type, males had a lower *Sarcocystis* positivity rate (22%; 95% CI 12–34%) compared to females (74%; 95% CI 63–84%). The estimated odds of *Sarcocystis* spp. infection in the male beef group was 0.10 (95% CI 0.04–0.21; *p* < 0.001) times smaller compared to female beef cattle, and the odds in female dairy carcasses was 3.5 (95% CI 1.4–10; *p* = 0.014) times larger than in female beef carcasses.

When considering the different muscles, *Sarcocystis* spp. were detected more often in the heart muscle than in the diaphragm among female beef carcasses (70% vs. 28%, McNemar test statistic = 26; df = 1, *p* < 0.001) and dairy carcasses (82% vs. 55%, *n* = 66; McNemar test statistic = 11, df = 1, *p* = 0.001; Fig. 2). Within male beef carcasses, no significant difference was observed (McNemar = 0.1, df = 1, *p* = 0.763).

### *Sarcocystis* spp. occurrence for different ages

**Fig. 3:**
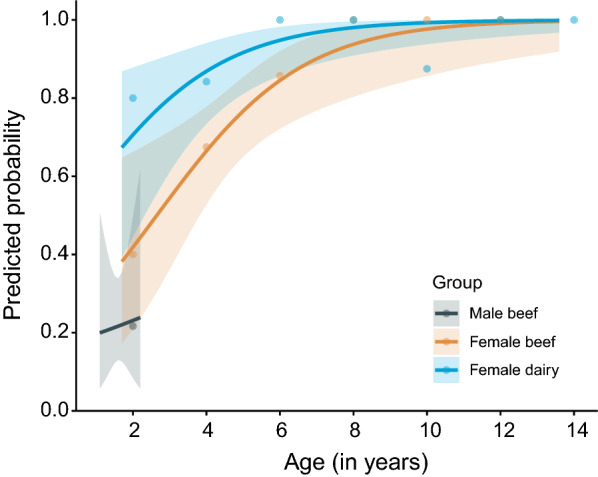
*Sarcocystis* positivity with age. The lines represent the predicted probabilities with the 95% confidence interval, in carcasses from male beef (*n* = 60), female beef (74) and female dairy cattle (*n* = 66). The points represent the observed proportions of *Sarcocystis*-positive carcasses, binned per 2 years and plotted at the midpoint of the age category (e.g. the points at 2 years represent the positivity rate of carcasses between 1 and 3 years within each of the groups)

Since carcasses from males (range from 1.1 to 2.2 years) were generally much younger than females (range from 1.7 to 13.6 years) (Fig. 1), the effect of age was evaluated separately for carcasses from male and female cattle. For female carcasses, the odds of finding sarcocysts significantly increased with age (OR_adjusted_ = 1.66 per year, 95% CI 1.22–2.41, *p* = 0.003) within both types (Fig. 3). The age-adjusted odds ratio for female dairy carcasses was 3.3 times higher (95% CI 1.24–10.3; *p* = 0.024) than for female beef carcasses. In female carcasses below 7 years old, *Sarcocystis* was observed in 71% (47/66) of the beef and 89% (39/44) of the dairy type, whereas all the female beef (8/8, 100%) and all but one dairy carcass (21/22, 95%) above 7 years old had a *Sarcocystis* infection (Fig. 3). Within the carcasses of male cattle, the effect of age was not significant (OR = 1.2 [0.1–15.5]; *p* = 0.872). The effect of age was also explored for presence of infection in diaphragm and heart samples separately and is visualized in Additional file 2.

### Intensity of sarcocysts

The number of sarcocysts in the 10 ml of sediment of the aliquot of the sample was recorded when conducting the microscopic examinations, censored at 15 sarcocysts. In *Sarcocystis*-positive heart samples for which the intensity was recorded (*n* = 104), the median intensity was the upper limit (i.e. 15 or above). In contrast, the median number of sarcocysts in positive diaphragm samples (*n* = 64) was three. The intensity of sarcocysts was similar in the different groups of cattle (data not shown).

### Species distribution

After microscopic examination of the sediment, 140 morphologically identified sarcocysts from 115 heart samples were further molecularly identified (one sarcocyst from 94 hearts, two sarcocysts from 18 hearts, three sarcocysts from two hearts and four sarcocysts from one heart). For diaphragm samples, 115 morphologically identified sarcocysts were further molecularly identified (one sarcocyst from 31 diaphragms, two sarcocysts from 25 diaphragms, three sarcocysts from 10 diaphragms and four sarcocysts from one diaphragm). Firstly, based on the mPCR of the morphologically identified sarcocysts, five sarcocysts from hearts and seven sarcocysts from diaphragms were *Sarcocystis* spp.-negative. In addition, a total of 28 sarcocysts could not be identified by mPCR at species level, thus they were subjected to PCR–RFLP for species identification. The species of 24 of these sarcocysts could not be identified by PCR–RFLP, and 22 of them were sent for *cox*1 sequencing. For two sarcocysts the *cox*1 PCR was negative, so these sarcocysts were considered *Sarcocystis*-positive, but the species was considered unidentified. After sequencing, the species of three sarcocysts could not be identified (Additional file 3), resulting in a total of five sarcocysts for which the species remained unidentified (two from the heart and three from the diaphragm).

**Fig. 4:**
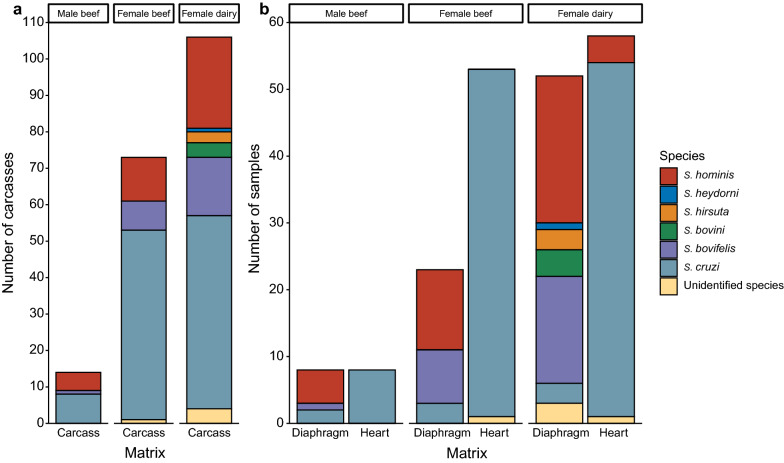
*Sarcocystis* species distribution (**a**) in carcasses and (**b**) in diaphragm/heart samples. Results are from *Sarcocystis*-positive carcasses of male beef (*n* = 13), female beef (*n* = 55) and female dairy cattle (*n* = 60). For each species, the height of the bar represents the number of positive carcasses/samples. The overall total number of positive samples does not correspond to the total number of *Sarcocystis*-positive carcasses/samples due to the presence of mixed infections (multiple species in one carcass/organ)

Overall, six different species were identified from the 200 carcasses (both organs combined; Fig. 4a). *Sarcocystis cruzi* was the most prevalent species and was found in 56.5% (113/200) of the carcasses, followed by *S. hominis* (21.0%, 42/200), *S. bovifelis* (12.5%, 25/200), *S. bovini* (2.0%, 4/200), *S. hirsuta* (1.5%, 3/200) and *S. heydorni* (0.5%, 1/200). In five carcasses (2.5%), unidentified *Sarcocystis* species were detected. Within each of the three cattle groups, *S. cruzi* was the most prevalent species, followed by *S. hominis* and *S. bovifelis* (Fig. 4a). *Sarcocystis heydorni*, *S. bovini* and *S. hirsuta* were found in female dairy cattle only.

In heart muscles, only two species, *S. cruzi* (56.5%, 113/200) and *S. hominis* (2.0%, 4/200), were identified, and for two sarcocysts the exact species could not be identified (Fig. 4b). Six *Sarcocystis* species were observed from diaphragm muscles. *Sarcocystis hominis* (19.5%, 39/200) had the highest infection rate, followed by *S. *bovifelis, *S. cruzi*, *S. bovini*, *S. hirsuta* and *S. heydorni*. For three diaphragm samples, the species could not be identified (Fig. 4b).

**Fig. 5:**
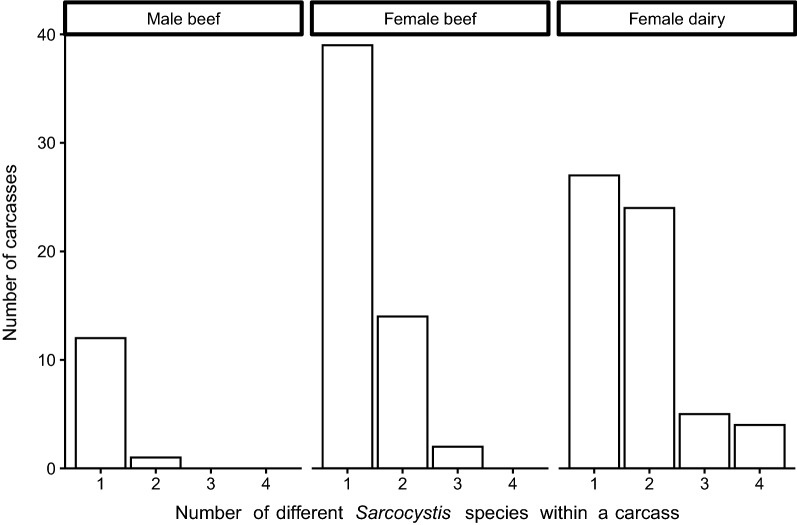
Number of different *Sarcocystis* species within a carcass (based on heart and diaphragm). Results are presented for *Sarcocystis* spp.-positive male beef (*n* = 13), female beef (*n* = 55) and female dairy carcasses (*n* = 60)

In 30% of the 128 *Sarcocystis*-infected carcasses (39/128), two different species were observed, and in 8.6% (11/128) of the carcasses, 3–4 different species were observed (Fig. 5). In the majority of the infected heart and diaphragm muscles, only one *Sarcocystis* species was observed (109 out of 114 positive heart samples, and 51 out of 64 positive diaphragm samples). In five heart muscles, two different species were observed, while in seven and six diaphragm muscles, two and three species were observed, respectively. Only in female dairy carcasses, up to four different species were detected within one carcass (*n* = 4 out of 60 *Sarcocystis*-positive carcasses; Fig. 5). The number of different species in female dairy carcasses was significantly higher than in male beef carcasses (*W* = 579, *p* = 0.007) and female beef cattle (*W* = 2120, *p* = 0.007). The difference in the number of different species between male beef and female beef carcasses was not statistically significant (*W* = 280, *p* = 0.111).

## Discussion

The final results in our study showed an overall prevalence of 64% in 200 cattle carcasses. Vangeel et al. [23] reported a 94% occurrence rate of *Sarcocystis* spp. in raw minced beef originating from retail stores in Belgium. This difference could be explained by the fact that minced meat is a mixture of meat from several carcasses as well as different muscles. The chances of finding *Sarcocystis* spp*.* in these ‘mixed’ samples is higher than in a single/separate sample from one carcass.

Muscles from the heart, tongue, esophagus and diaphragm are the target-colonizing areas of *Sarcocystis* spp*.* [1]. In the present study, a significantly higher occurrence rate was observed in heart (57%) than in diaphragm (32%) samples. Similarly, Yang et al. [18] also reported more *Sarcocystis* spp. in heart muscles (49%) than in diaphragms (14%). In the study of Latif et al. [16], the occurrence rate in the heart (8%) was lower than in the diaphragm (27%) in cattle, while in water buffaloes, the heart was the most often infected organ (67%), with no *Sarcocystis* spp*.* detected in the diaphragm. High occurrence rates, ranging between 58% and 99.5% in hearts and 58%–90.9% in diaphragms in cattle, have been reported from different countries, e.g. Iran [19, 35], Italy [22], the Netherlands [24], Brazil [17] and Argentina [36]. This shows that although heart and diaphragm are target-colonizing areas of *Sarcocystis* spp., the occurrence rate can vary between countries/studies. Besides the differences between organs/muscles mentioned above, also differences in detection and identification techniques used (e.g. morphological identification versus molecular identification) and differences in study populations (e.g. age, sex, breed), render comparison between the studies complicated.

Differences in breed as well as sex and age of the host, climatic factors and management practices [37] may all result in differences in the prevalence estimates of *Sarcocystis* spp*.* in cattle. This was also highlighted in our study, where the *Sarcocystis* spp*.* occurrence rate differed markedly between the different groups of animals, with the highest rate observed in female dairy, followed by female beef, and the lowest in male beef cattle. In contrast to our results, Savini et al. [37] found the highest prevalence of *Sarcocystis* spp. infections in esophagus samples of entire males (92%) (*n* = 51) compared to either castrated males (60%) (*n* = 261) or females (51%) (*n* = 170). On the other hand, Hornok et al. [21] found no significant difference between infection rates of individually-collected heart and esophagus samples from bulls (67%) and cows (64%). However, the mean age of PCR-positive cattle (6.2 ± 4.4 years) was significantly higher than that of PCR-negative ones (4.7 ± 4 years). Similarly, we also found a significant increase in *Sarcocystis* infections with increasing age for the carcasses of beef and dairy cows. This increase was partially similar to the study of Savini et al. [37], in which an increasing prevalence of *Sarcocystis* spp. infection was found in cattle up to the age of 4 years, after which the prevalence significantly decreased. Savini et al. [37] hypothesized that this might be explained by the reduction of sarcocysts over time because of the host response or a reflection of (natural or managerial) selection, where the generally healthier animals are kept for longer periods. In the present study, we did not observe such a decrease, as the vast majority of animals above 7 years were infected with *Sarcocystis* species. Savini et al. [37] attributed the increased prevalence with age (until 4 years) to the greater opportunity for older animals to be exposed to oocysts/sporocysts. In Belgium, dairy cattle are often kept outdoors, permitting contact with potentially contaminated water and soil/pasture. Additionally, dairy cattle are generally kept longer than beef cattle, allowing more exposure time, and leading to an older age when arriving at the slaughterhouse. Indeed, the higher occurrence of *Sarcocystis* spp. in carcasses of dairy cows than of beef cows was partially explained by the higher slaughter age in the former group. Since the bulls in the present study were slaughtered at a considerably younger age than the cows, we did not want to extrapolate the age effect from cows to bulls. Nevertheless, we believe that the lower slaughter age of male beef cattle may also (partially) explain the lower occurrence rate in carcasses of bulls in our study. Still, it is obvious that other factors besides age may have contributed to the differences between groups, since carcasses of dairy cows still had a significantly higher odds of being infected than beef cows after correcting for the age effect. We did not collect/evaluate other variables, such as the breed, feed, outdoor access and geographical information during this study as this was not our main aim, but future research studying such factors should be encouraged, as an increased epidemiological understanding of these parasites may contribute to the control of *Sarcocystis* infections in cattle.

Six *Sarcocystis* species were identified through molecular tools, i.e. *S. cruzi*, *S. hominis*, *S. bovifelis*, *S. hirsuta*, *S. heydorni* and *S. bovini*. *Sarcocystis cruzi* followed by *S. hominis* had the highest prevalence among cattle, which was similar with the study of Hoeve-Bakker et al. [24] and Hornok et al. [21]. Significantly more different species were observed in our female dairy group. This might be explained by longer exposure times (outdoor access, age) of this specific cattle type. Interestingly, when looking into different muscles, the diaphragm had a higher species diversity (*S. cruzi*, *S. hominis*, *S. bovifelis*, *S. hirsuta*, *S. heydorni* and *S. bovini* and unidentified species) than the heart (*S. cruzi*, *S. hominis* and unidentified species). *Sarcocystis cruzi* was the most prevalent species in the heart, while in the diaphragm this was *S. hominis*. *Sarcocystis hominis* and *S. heydorni* are zoonotic *Sarcocystis* species and can cause intestinal sarcocystiosis in humans [7]. When *S. hominis*- and/or *S. heydorni*-infected diaphragms enter the food chain, they might pose a high risk for public health, depending on the further processing. In the Belgian study of Vangeel et al. [23], 63 of 67 minced meat samples were found infected, with thick-walled sarcocysts detected in 61 samples. Molecular analyses (including PCR and sequencing) of the latter samples could reliably identify 39 PCR products, of which 38 were confirmed as *S. hominis*. This seems much higher than the occurrences observed in the current study. Yet again, results are difficult to compare as minced meat is composed of several organs of several carcasses.

## Conclusions

In this study, *Sarcocystis* spp. were detected in 64% of sampled carcasses. Nevertheless, extrapolation of these results to the Belgian cattle population at slaughter point should be done carefully, as the sampled population does not fully represent the total population. Indeed, the conformation of R in the dairy type was slightly oversampled compared to the total population of slaughtered cattle in Flanders. Moreover, beef cattle in Flanders are rather specific, with its typical Belgian White Blue breed that is characterized by muscular hypertrophy (and related management practices), which may limit generalizability of our data to other cattle populations. Significantly more infections were detected in female dairy cattle (91%), and the odds of infection increased with age in female animals. More infections were detected in the heart muscles, where *S. cruzi* was the most prevalent species, while in the diaphragm, this was *S. hominis*. The detection of *S. hominis* in 21% of the sampled carcasses presents a potential food safety issue, and further research is warranted into controlling this infection.

## Supplementary Information

**Additional file 1:** Expected fragment sizes (in base pairs) from digestion of 18S rDNA PCR products of bovine *Sarcocystis* species with *Fok*I and *Bfa*I.

**Additional file 2:** *Sarcocystis* positivity rate in diaphragm and heart samples among different groups.

**Additional file 3:** Phylogenetic tree of unidentified *Sarcocystis* species (*cox*1 sequencing).

## Data Availability

The dataset supporting the conclusions of this article is available in the Zenodo repository, https://doi.org/10.5281/zenodo.4611489. The nucleotide sequences of the unidentified *Sarcocystis* spp. UNS1, UNS2, UNS3 are in GenBank under the following accession numbers: UNS1: MW756133, UNS2: MW756134, UNS3: MW756135.

## Abbreviations

AIC: Akaike information criterion
BEM: Bovine eosinophilic myositis
bp: Base pairs
CI: Confidence interval
*cox*1: Cytochrome c oxidase subunit I gene
mPCR: Multiplex polymerase chain reaction
OR: Odds ratio
RFLP: Restriction fragment length polymorphism
