# Supplementary material for: Sarcocystis species in bovine carcasses from a Belgian abattoir: a cross-sectional study
Source: Parasit Vectors. 2021 May 21;14:271. doi: 10.1186/s13071-021-04788-1 (PMC8138977; doi:10.1186/s13071-021-04788-1)
Supplement: Supplementary file 1 — Additional file 1: Expected fragment sizes (in base pairs) from digestion of 18S rDNA PCR products of bovine Sarcocystis species with FokI and BfaI. [file 13071_2021_4788_MOESM1_ESM.pdf]

Additional File 1. Expected fragment sizes (in base pairs) from digestion of 18S rDNA PCR products of bovine *Sarcocystis* species with *Fok*I and *Bfa*I

	FokI	BfaI
S. cruzi	321, 535 [1-3]	108, 360, 388 [1-3]
S. hirsuta	77, 81, 207, 518 [1-6]	**403, 480 [1-4]** (or 210, 270, 403 [5] or 108, 372, 402 [6])*
S. bovifelis[#]	/ [1-5]	**108, 353, 380 [1-3]** (or 30, 108, 350, 356 [4] or 70, 108, 309, 359 [5])
S. bovini[#]	/ [1-6]	**108, 356, 380 [1-4]** (or 108, 145, 214, 379 [5] or 380, 464 [6])
S. hominis	**/ [1-3]** (or 324, 528 [4])	382, 474 [1-4]
S. heydorni[#]	/ [1, 2]	108, 368, 389 [1, 2]

Small numbers mark the corresponding gene sequences with accession numbers.

S. cruzi (1: JX679467; 2: AF017120; 3: KT901167)

S. hirsuta (1: KT901166; 2: KC209741; 3: JX855283; 4: KT901158; 5: KT901157; 6: AF017122)

S. bovifelis (1: KT901138; 2: KT901130; 3: KT901123; 4: KT901117; 5: KT901131)

S. bovini (1: KT901139; 2: KT901140; 3: KT901142; 4: KT901155; 5: KT901146; 6: KT901141)

S. hominis (1: JX679470; 2: AF176944; 3: KF954731; 4: JX679471)

S. heydorni (1: KX057996; 2: KX057997)

/ = No digestion

[#] *S. bovini*, *S. bovifelis* and *S. heydorni* cannot be unambiguously differentiated from each other by the 18S rDNA PCR-RFLP.

*Digestion profiles shown in brackets are possible but rare.
