# Supplementary material for: Sarcocystis species in bovine carcasses from a Belgian abattoir: a cross-sectional study
Source: Parasit Vectors. 2021 May 21;14:271. doi: 10.1186/s13071-021-04788-1 (PMC8138977; doi:10.1186/s13071-021-04788-1)
Supplement: Supplementary file 2 — Additional file 2: Sarcocystis positivity rate in diaphragm and heart samples among different groups. [file 13071_2021_4788_MOESM2_ESM.pdf]

Additional file 2. *Sarcocystis* positivity rate in (A) heart and (B) diaphragm samples among different age categories

A) Heart

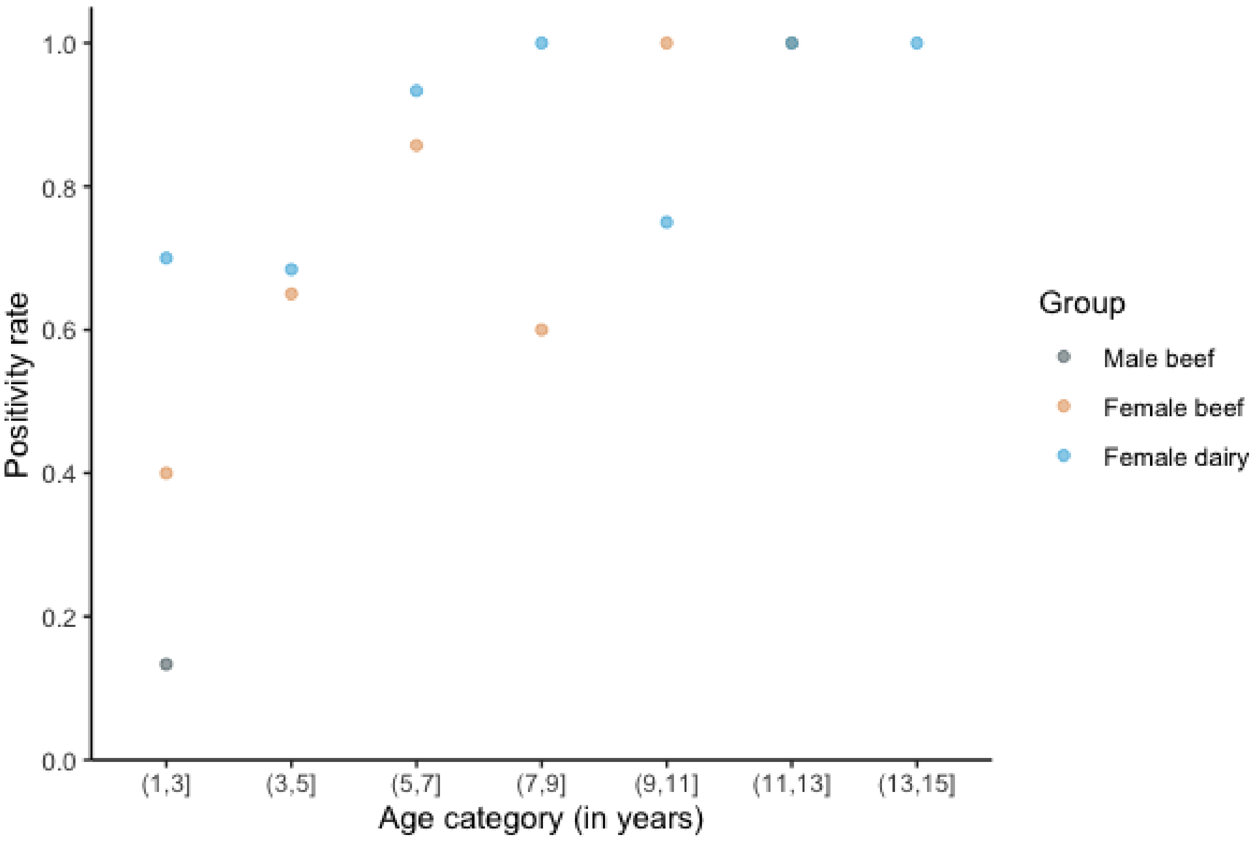

B) Diaphragm

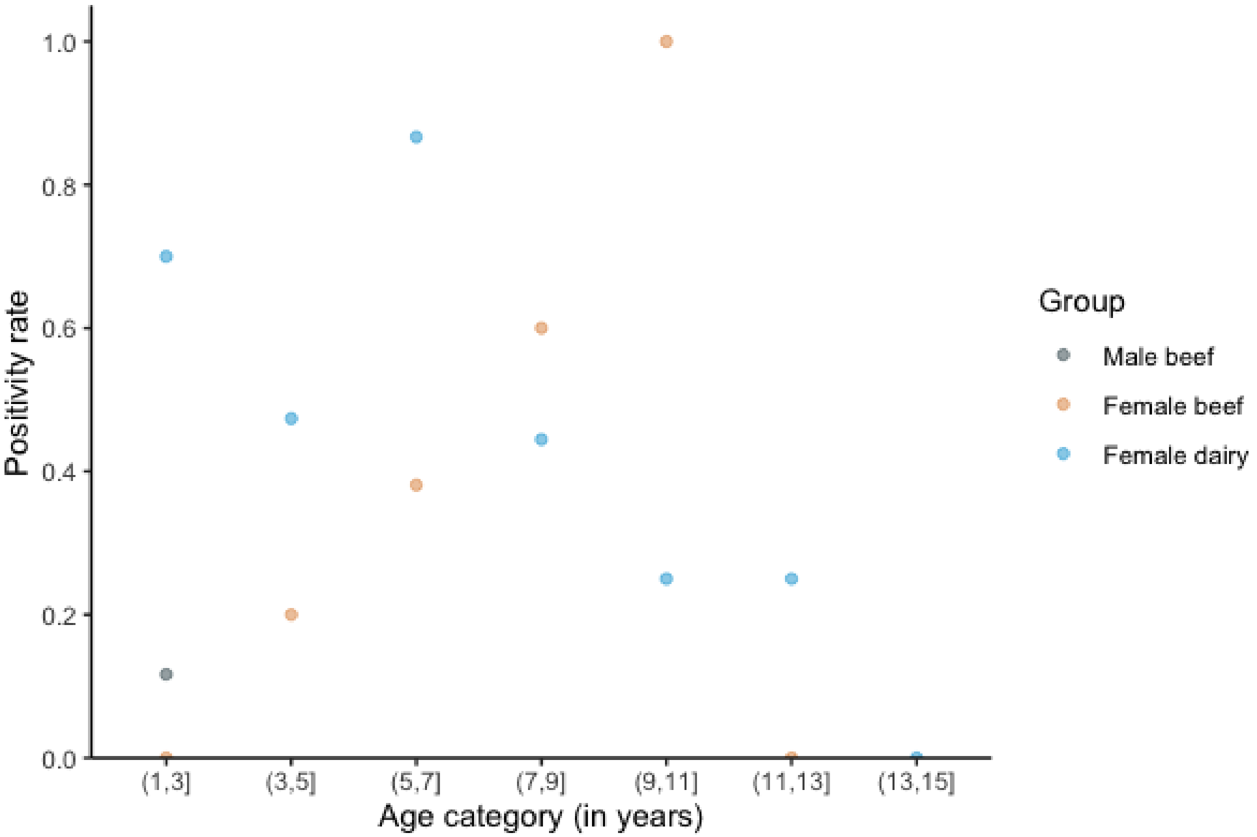

Positivity rate
1.0
0.8
0.6
0.4
0.2
0.0
(1,3]
(3,5]
(5,7]
(7,9]
(9,11]
(11,13]
(13,15]
Age category (in years)
Group
Male beef
Female beef
Female dairy
