# Supplementary material for: Sarcocystis species in bovine carcasses from a Belgian abattoir: a cross-sectional study
Source: Parasit Vectors. 2021 May 21;14:271. doi: 10.1186/s13071-021-04788-1 (PMC8138977; doi:10.1186/s13071-021-04788-1)
Supplement: Supplementary file 3 — Additional file 3: Phylogenetic tree of unidentified Sarcocystis species (cox1 sequencing). [file 13071_2021_4788_MOESM3_ESM.pdf]

Additional File 3. Phylogenetic tree of unidentified *Sarcocystis* species (*cox*1 sequencing).

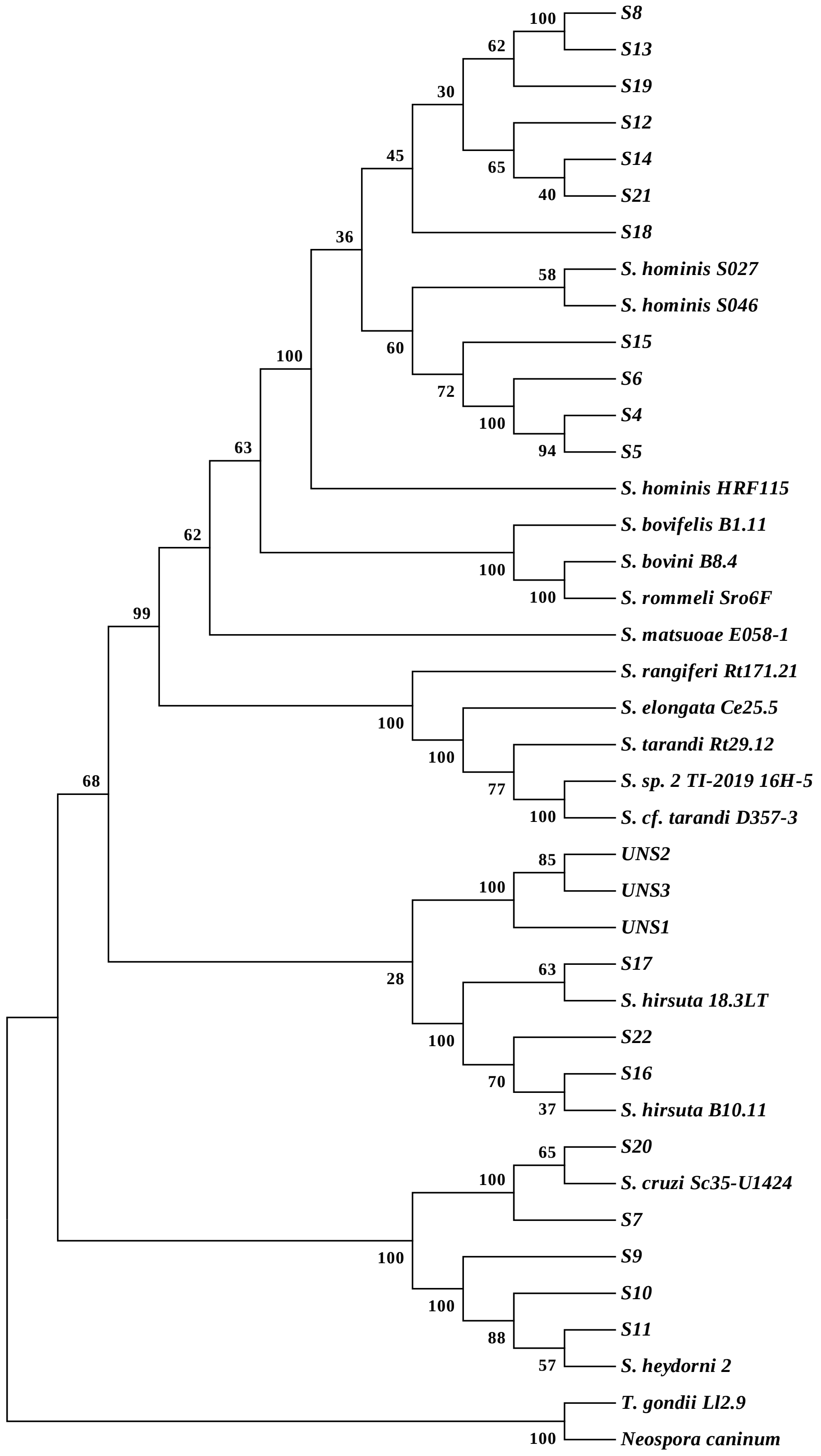

Additional file 3. Phylogenetic tree of unidentified *Sarcocystis* species (*cox*1 sequencing).

The phylogenetic tree was constructed with MEGA 7.0 using the neighbor-joining method. Bootstrap values of 1000 replications (%) were indicated on the branches. Three unidentified (UNS1, UNS2, UNS3) and nineteen identified (S4-22) *Sarcocystis* species via *cox*1 sequencing sequences and eighteen sequences covered different representative species were selected from the BLAST results, and two outer groups (*T. gondii* LI2.9 and *Neospora caninum*) were selected to generate a phylogenetic tree.

The species are listed below: *S. hominis* S046: MK497843.1, *S. hominis* S027: MK497842.1, *S. hominis* HRF115: MK497840.1, *S. bovifelis* B1.11: KT900962.1, *S. bovini* B8.4: KT901016.1, *S. rommeli* clone Sro6F: KY120292.1, *S. matsuoae* E058-1: LC481076.1, *S. rangiferi* Rt171.21: KF241407.1, *S. elongata* Ce25.5: KF241324.1, *S. tarandi* Rt29.12: KF241413, *S.* sp. 2 TI-2019 16H-5: LC466192, *S. cf. tarandi* D357-3: LC481070.1, *S. hirsuta* 18.3LT: MT796947.1, *S. hirsuta* B10.11: KT901052.1, *S. cruzi* Sc35-U1424: LC171861.1, *S. heydorni* 2: KX057995.1, *T. gondii* Ll2.9: KM657810.1, *Neospora caninum*: MN077085.1. The GenBank accession numbers of nucleotide sequences of UNS1, UNS2, UNS3 are as follows: UNS1: MW756133, UNS2: MW756134, UNS3: MW756135.

Among them, UNS1 was most closely related to species *S. bovini* B8.4 with an identity of 82.98%, UNS2 was *S. bovini* B8.4 with an identity of 83.19%, UNS3 was *S. bovini* B8.4 with an identity of 83.01%, S4 was *S. hominis* S027 with an identity of 98.65%, S5 was *S. hominis* S027 with an identity of 98.44%, S6 was *S. hominis* S027 with an identity of 98.96%, S7 was *S. cruzi* Sc35-U1424 with an identity of 97.53%, S8 was *S. hominis* HRF115 with an identity of 98.24%, S9 was *S. heydorni* with an identity of 99.00%, S10 was *S. heydorni* with an identity of 99.80%, S11 was *S. heydorni* with an identity of 98.91%, S12 was *S. hominis* HRF115 with an identity of 98.16%, S13 was *S. hominis* HRF115 with an identity of 98.04%, S14 was *S. hominis* HRF115 with an identity of 98.26%, S15 was *S. hominis* S027 with an identity of 98.44%, S16 was *S. hirsuta* B10.11 with an identity of 99.69%, S17 was *S. hirsuta* 18.3LT with an identity of 99.20%, S18 was *S. hominis* 046 with an identity of 97.61%, S19 was *S. hominis* HRF115 with an identity of 97.24%, S20 was *S. cruzi* Sc35-U1424 with an identity of 98.45%, S21 was *S. hominis* HRF115 with an identity of 97.96%, S22 was *S. hirsuta* B10.11 with an identity of 99.80%.

Additional file 3. Phylogenetic tree of unidentified *Sarcocystis* species (*cox*1 sequencing).
